# Towards the elimination of tuberculosis in Latin America: opportunities through network cooperation

**DOI:** 10.1590/1518-8345.0000.4575

**Published:** 2025-08-18

**Authors:** Erica Chimara, Pedro Almeida Silva, Kleydson Bonfim Andrade, Andrea Villarino, Gabriela Gago, Ricardo Alexandre Arcêncio

**Affiliations:** 1 Instituto Adolfo Lutz, Núcleo de Tuberculose e Micobacterioses, São Paulo, SP, Brazil.; 2 Rede Brasileira de Pesquisas em Tuberculose, Rio de Janeiro, RJ, Brazil.; 3 Universidade Federal do Rio Grande, Rio Grande, RS, Brazil.; 4 Organização Pan-Americana de Saúde, Brasília, DF, Brazil.; 5 Universidad de la República, Facultad de Ciencias, Montevideo, Uruguay.; 6 Sociedad Latinoamericana de Tuberculosis y otras micobacteriosis.Uruguay.; 7 Instituto de Biología Molecular y Celular de Rosario, Santa Fe, Argentina.; 8 Universidade de São Paulo, Escola de Enfermagem de Ribeirão Preto, PAHO/WHO Collaborating Centre for Nursing Research Development, Ribeirão Preto, SP, Brazil.

**Figure:**
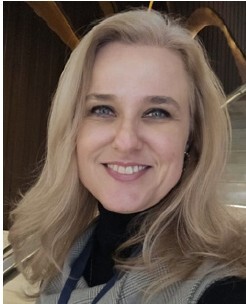

**Figure:**
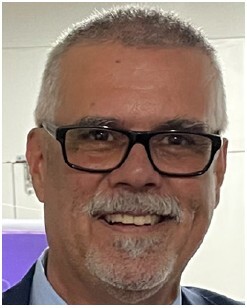

**Figure:**
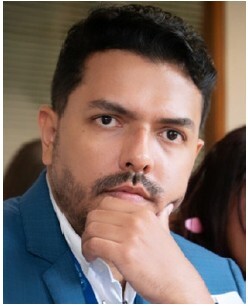

**Figure:**
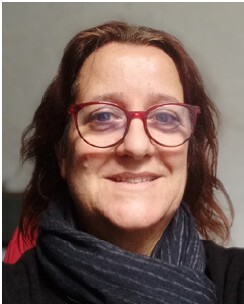

**Figure:**
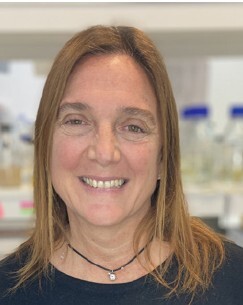

**Figure:**
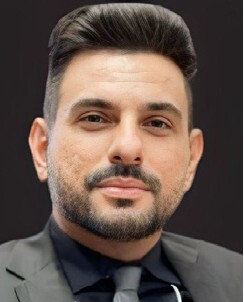

The latest World Health Organization report highlights the alarming situation of tuberculosis (TB) in Latin America (LA). Between 2020 and 2023, a significant increase in mortality (14%) and a slight decrease in 2024 (13%)^(1)^ was observed in LA. This article aims to analyze this problem and highlight the importance of forming a TB Research Network for the region in order to achieve the WHO objective of ending TB in LA.

The countries of LA face multiple challenges that hinder a more accelerated progress towards TB elimination. These include socio-economic, cultural, educational and health service organization factors. Thus, problems such as TB/HIV coinfection, the increase in diabetes, smoking and other chronic diseases are added to factors such as malnutrition, the use of psychoactive substances and limited access to health services. These factors are accentuated by social and economic inequalities in the region, which make it even more difficult to eliminate TB in LA.

Another cause for concern is the increase in cases of multidrug-resistant TB (MDR-TB) and extensively drug-resistant TB (XDR-TB), highlighting their heterogeneous distribution, which varied in 2023 between 0-11.9% of all new TB cases, depending on the LA country^(1)^. These cases represent a critical challenge for health systems and patients due to their long-term treatment, which can exceed 20 months, and the risk of transmission of resistant strains. However, it is worth noting that several countries in the region have already implemented shortened six-month oral BPaL and BPaLM regimens for the treatment of RR/MDR and pre-XDR tuberculosis.

Furthermore, the economic impact is considerable. While treating a case of sensitive TB costs between US$100 and US$500, MDR-TB treatment ranges from US$5,000 to US$15,000, and for XDR-TB it can exceed US$30,000 per patient^(2)^. While the region can count on the assistance of the Pan American Health Organization’s strategic fund to subsidize the costs of these treatments, much work remains to be done to ensure that all patients have access. This panorama reinforces the urgent need to strengthen strategies for prevention, early diagnosis and adequate management of drug resistance in the region.

Early diagnosis of TB in many LA countries faces serious difficulties due to the insufficient availability of resources for rapid diagnosis, either through laboratory techniques or imaging, and insufficient training of health professionals. However, it is important to remark that LA has the best levels of bacteriological confirmation globally, reaching an average of 86% in 2023. This indicator reflects the region’s improved capacity for timely diagnosis with currently available methods, which represents an important advance in the fight against TB^(1)^. Nonetheless, it is still necessary to expand the active search focused on the most vulnerable populations and areas affected by TB.

In 2023, the 78th United Nations General Assembly adopted the political declaration “End TB”, a global commitment that highlights the need to advance science, financing and innovation, as well as the benefits of the urgent combat against the global TB epidemic. This declaration emphasizes ensuring equitable access to prevention, diagnostic testing, treatment and dignified care for all affected people^(3)^. The End TB Strategy highlights networking as one of the main approaches to achieve the goal of ending TB as an epidemic by 2035 and eliminating it completely by 2050^(4)^. The approach involves a comprehensive and interdisciplinary collaboration that brings together actors such as governments, non-governmental organizations, industry, health professionals, researchers, managers and civil society^(5)^.

**Figure 1-:**
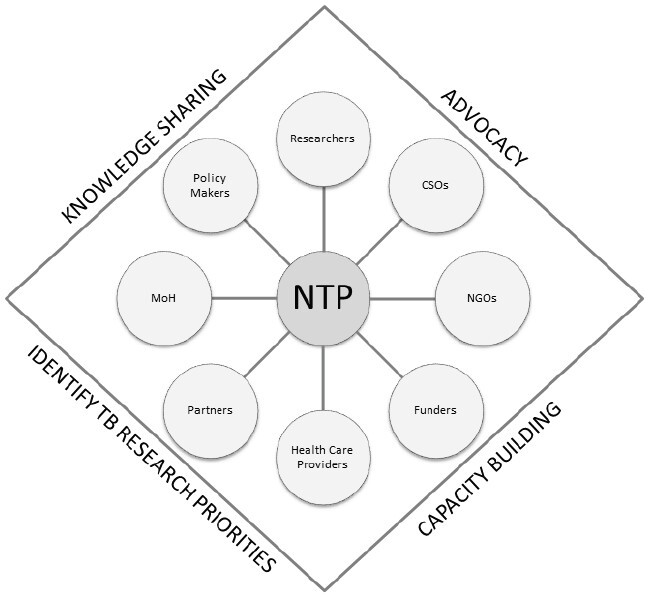
Fundamental objectives of a TB Cooperative Network

Source: https://new.aidsdatahub.org/sites/default/files/resource/toolkit-developing-national-tb-research-plan-2016.pdf

Networking is structured around four central objectives^(5)^ which are: 1. Strengthening comprehensive and focused care for people affected by TB; 2. Intensifying public policies and support systems related to TB, 3. Promoting research and innovation to improve diagnostic, therapeutic and preventive tools and 4. Ensuring financial sustainability and long-term political commitment to eradicate the disease (Figure 1).

NTP = National Tuberculosis Program; CSO = Civil society organization; NGO = Non-governamental organizations; MoH = Ministry of Health

Several initiatives have been described at the international level to fulfill this purpose, amongst which the following stand out:

**European Research Initiative on Tuberculosis (ERI-TB):** Focused on ensuring the research and innovation necessary for the elimination of TB in Europe.

**TB BRICS Research Network:** Launched in 2017, this network includes Brazil, Russia, India, China and South Africa, and more recently added Iran, Egypt and Ethiopia.

**Ethiopian Tuberculosis and Research Advisory Committee (TRAC):** Focused on strengthening research capacity and strategies to address TB in specific contexts.

**Vietnam Integrated Tuberculosis and Respirology Research Center (VICTORY):** A key research and development initiative in Asia aimed at the prevention and treatment of respiratory diseases, including TB.

These initiatives reflect the global commitment to fight against TB, promoting collaboration between regions and countries to achieve innovative and effective solutions^(5)^.

If we focus on LA, there are precedents of regional cooperation. For example, in Brazil, the Brazilian Tuberculosis Research Network (REDE-TB) initiative, launched in 2001, stands out as a key initiative in the promotion of research, scientific collaboration and articulation with the national TB control program. Other similar initiatives that connect research and clinical research exist in Colombia, Chile, Mexico and Peru, but there is no connection between them. In addition, in 2006, the Latin American Society of Tuberculosis and other mycobacteriosis (SLAMTB) was consolidated, a network of Latin American scientists that has contributed to the promotion, information and scientific collaboration on TB, without greater political coordination with other actors and strategies to comply with the four central points of a network.

The establishment of a broad, integrated research network in LA is expected to have a significant impact on progress toward TB elimination. One example of the value of scientific collaboration is a study that showed that countries with higher rates of international collaboration are able to boost the quantity and quality of scientific and technological production^(6)^.

The exchange of knowledge not only encourages joint work between researchers, but also promotes the development of more effective methods for the prevention, diagnosis and treatment of TB. The lack of an integrated approach that combines strategic actors, such as government, academia and civil society, hinders progress in the fight against the disease and underlines the urgent need for a solid and coordinated regional network. It is essential to adopt an integrated and coordinated approach that goes beyond the health sector, also involving education, community participation, social policies and international cooperation.

This collaborative network will maximize available resources, improve equitable access to care, raise the quality of services, and promote research and innovation. In this way, we will be able to create an enabling environment for TB research and innovation, increase funding for research and innovation in the region, promote and improve approaches to data sharing, and ensure equitable access to the benefits of research and innovation. With the support of the Pan American Health Organization and the participation of all countries in the region, the comprehensive response to TB will be strengthened, respecting the particularities and capacities of each nation. This joint effort reaffirms LA’s commitment to a future where TB is eliminated and health equity is a reality.
