# Supplementary material for: Towards the elimination of tuberculosis in Latin America: opportunities through network cooperation
Source: Rev Lat Am Enfermagem. 2025 Aug 18;33:e4575. doi: 10.1590/1518-8345.0000.4575 (PMC12364453; doi:10.1590/1518-8345.0000.4575)
Supplement: Translation

# Translation: Rumo à eliminação da tuberculose na América Latina: oportunidades por meio da cooperação em rede

**DOI:** 10.1590/1518-8345.0000.4576

**Authors:** Erica Chimara, Pedro Almeida Silva, Kleydson Bonfim Andrade, Andrea Villarino, Gabriela Gago, Ricardo Alexandre Arcêncio

**Affiliations:** 1 Instituto Adolfo Lutz, Núcleo de Tuberculose e Micobacterioses, São Paulo, SP, Brasil.; 2 Rede Brasileira de Pesquisas em Tuberculose, Rio de Janeiro, RJ, Brasil.; 3 Universidade Federal do Rio Grande, Rio Grande, RS, Brasil.; 4 Organização Pan-Americana de Saúde, Brasília, DF, Brasil.; 5 Universidad de la República, Facultad de Ciencias, Montevideo, Uruguai.; 6 Sociedad Latinoamericana de Tuberculosis y otras micobacteriosis. Uruguai.; 7 Instituto de Biología Molecular y Celular de Rosario, Santa Fe, Argentina.; 8 Universidade de São Paulo, Escola de Enfermagem de Ribeirão Preto, Centro Colaborador da OPAS/OMS para o Desenvolvimento da Pesquisa em Enfermagem, Ribeirão Preto, SP, Brasil.

O último relatório da Organização Mundial da Saúde destaca a situação alarmante da tuberculose (TB) na América Latina (AL). Entre 2020 e 2023, observa-se um aumento significativo da mortalidade (14%) na AL e uma ligeira diminuição em 2024 (13%)^(1)^. Este editorial tem como objetivo analisar esse problema e destacar a importância da criação de uma Rede de Pesquisa em TB para a região para atingir a meta da OMS de acabar com a TB na AL.

Os países latino-americanos enfrentam vários desafios que impedem um progresso mais rápido em direção à eliminação da TB. Isso inclui fatores socioeconômicos, culturais, educacionais e de organização de serviços de saúde. Problemas como a coinfecção TB/HIV, o aumento da diabetes, tabagismo e outras doenças crônicas somam-se a fatores como a desnutrição, o consumo de substâncias psicoativas e o acesso limitado aos serviços de saúde. Fatores que são acentuados pelas desigualdades sociais e econômicas da região, tornando ainda mais difícil a eliminação da TB na AL.

Outro aspecto preocupante é o aumento de casos de TB multirresistente (TB-MDR) e TB extensivamente resistente (TB-XDR), destacando sua distribuição heterogênea, que variou em 2023 entre 0-11,9% dos novos casos de TB de acordo com o país da AL^(1)^. Esses casos representam um desafio crítico para os sistemas de saúde e para os pacientes devido ao seu tratamento prolongado, que pode exceder 20 meses, e ao risco de transmissão de cepas resistentes, embora já tenhamos os regimes BPaL/BPaLM orais encurtados de seis meses de duração para RR /MDR e preXDR em vários países da Região.

Além disso, o impacto econômico é considerável. Enquanto o tratamento de um caso de TB suscetível custa entre 100 e 500 dólares, o tratamento para a TB-MR varia entre 5.000 e 15.000 dólares, e para a TB-XDR pode exceder os 30.000 dólares por paciente^(2)^. Embora a região possa contar com a ajuda do fundo estratégico da Organização Panamericana de Saúde (OPAS) para subsidiar os custos desses tratamentos, ainda há muito trabalho a ser feito para garantir que os pacientes tenham acesso. Este cenário reforça a necessidade urgente de fortalecer estratégias de prevenção, diagnóstico precoce e manejo adequado da resistência aos medicamentos na região.

O diagnóstico precoce da TB em muitos países da AL enfrenta sérias dificuldades devido à disponibilidade insuficiente de recursos para diagnóstico rápido, seja por meio de técnicas laboratoriais ou de imagem, e à formação insuficiente dos profissionais de saúde. Entretanto, é importante destacar que a AL apresenta os melhores níveis de confirmação bacteriológica do mundo, atingindo uma média de 86% em 2023. Este indicador reflete a melhora na capacidade da região de realizar diagnósticos oportunos com os métodos atualmente disponíveis. Isso representa um avanço importante no combate à TB^(1)^, mas ainda é necessária a ampliação da busca ativa voltada às populações em situação de maior vulnerabilidade e áreas afetadas pela TB.

Em 2023, durante a 78ª Assembléia Geral das Nações Unidas, foi aprovada a declaração política “Fim da TB”, um compromisso global que destaca a necessidade de avançar na ciência, no financiamento e na inovação, bem como os seus benefícios para combater de maneira urgente a epidemia mundial de TB. Esta declaração enfatiza a garantia de acesso equitativo à prevenção, aos testes de diagnóstico, ao tratamento e aos cuidados dignos para todas as pessoas afetadas^(3)^. A Estratégia pelo Fim da TB destaca a criação de redes como uma das principais abordagens para atingir o objetivo de mitigar a TB como epidemia até 2035 e eliminá-la completamente até 2050^(4)^. A abordagem envolve uma colaboração abrangente e interdisciplinar que reúne atores como governos, organizações não governamentais, indústria, profissionais de saúde, pesquisadores, gestores e sociedade civil^(5)^.

O trabalho em rede está estruturado em torno de quatro objetivos centrais^(5)^ que são: 1. Fortalecer a atenção integral focada nas pessoas afetadas pela TB; 2. Fortalecer as políticas públicas e os sistemas de apoio relacionados à TB, 3. Promover a pesquisa e a inovação para melhorar as ferramentas de diagnóstico, terapêuticas e preventivas e 4. Garantir a sustentabilidade financeira e o compromisso político de longo prazo para erradicar a doença (Figura 1).

**Figura 1-:** Objetivos fundamentais de uma Rede Cooperativa de TB

Fonte: https://new.aidsdatahub.org/sites/default/files/resource/toolkit-developing-national-tb-research-plan-2016.pdf

NTP = Programa Nacional de Tuberculose; CSO = Organização da sociedade civil; NGO = Organizações não governamentais; MoH = Ministério da Saúde

Várias iniciativas têm sido descritas em nível internacional para atingir este propósito, entre as quais se destacam:

**Iniciativa Europeia de Pesquisa sobre Tuberculose (ERI-TB)**: Focada em garantir a pesquisa e a inovação necessárias para eliminar a TB na Europa.

**Rede de Pesquisa sobre TB do BRICS**: Lançada em 2017, esta rede inclui Brasil, Rússia, Índia, China e África do Sul e, mais recentemente, incorporou Irã, Egito e Etiópia.

**Comitê Consultivo Etíope sobre Tuberculose e Pesquisa (TRAC):** Focado no fortalecimento da capacidade de pesquisa e estratégias para abordar a TB em contextos específicos.

**Centro de Pesquisa Integrada do Vietnã para Tuberculose e Respirologia (VICTORY):** Uma importante iniciativa de pesquisa e desenvolvimento na Ásia, visando a prevenção e o tratamento de doenças respiratórias, incluindo a TB.

Estas iniciativas refletem o compromisso global na luta contra a TB, promovendo a colaboração entre regiões e países para alcançar soluções inovadoras e eficazes^(5)^.

Se nos concentrarmos na AL, há antecedentes de cooperação regional. Por exemplo, no Brasil, a iniciativa da Rede Brasileira de Pesquisas em Tuberculose (REDE-TB), lançada em 2001, destaca-se como uma iniciativa fundamental na promoção da pesquisa, colaboração científica e articulação com o programa nacional de controle da TB. Outras iniciativas semelhantes que conectam pesquisa e prática clínica existem na Colômbia, Chile, México e Peru, mas não há conexão entre elas. Além disso, em 2006 foi consolidada a Sociedade Latino-Americana de Tuberculose e outras micobacterioses (SLAMTB), uma rede de cientistas da AL que tem contribuído para a promoção, informação e colaboração científica em torno da TB, sem maior coordenação política com outros atores e estratégias para cumprir com os quatro pontos centrais de uma Rede.

Espera-se que o estabelecimento de uma rede de pesquisa ampla e integrada na AL tenha um impacto significativo no progresso em direção à eliminação da TB. Um exemplo do valor da colaboração científica é um estudo que mostrou que países com maiores taxas de colaboração internacional são capazes de impulsionar a quantidade e a qualidade da produção científico-tecnológica^(6)^.

O compartilhamento de conhecimento não apenas incentiva o trabalho colaborativo entre pesquisadores, mas também impulsiona o desenvolvimento de métodos mais eficazes para a prevenção, diagnóstico e tratamento da TB. A falta de uma abordagem integrada que combine atores estratégicos, como governo, academia e sociedade civil, dificulta o progresso na luta contra a doença e ressalta a necessidade urgente de uma rede regional forte e coordenada. É essencial adotar uma abordagem integrada e coordenada que vá além do setor da saúde, envolvendo também educação, participação comunitária, políticas sociais e cooperação internacional.

Esta rede colaborativa maximizará os recursos disponíveis, melhorará o acesso equitativo aos cuidados, aumentará a qualidade dos serviços e promoverá a pesquisa e a inovação. Dessa forma, seremos capazes de criar um ambiente propício para a pesquisa e inovação em TB, aumentar o financiamento para pesquisa e inovação na região, promover e melhorar abordagens para o intercâmbio de dados e garantir acesso equitativo aos benefícios da pesquisa e inovação. Com o apoio da OPAS e com a participação de todos os países da região, a resposta integral à TB será fortalecida, respeitando as particularidades e capacidades de cada nação. Este esforço conjunto reafirma o compromisso da AL com um futuro onde a TB será eliminada e a equidade em saúde será uma realidade.

# Translation: Hacia la eliminación de la tuberculosis en América Latina: oportunidades a través de la cooperación en Red

**DOI:** 10.1590/1518-8345.0000.4574

**Authors:** Erica Chimara, Pedro Almeida Silva, Kleydson Bonfim Andrade, Andrea Villarino, Gabriela Gago, Ricardo Alexandre Arcêncio

**Affiliations:** 1 Instituto Adolfo Lutz, Núcleo de Tuberculose e Micobacterioses, São Paulo, SP, Brasil.; 2 Rede Brasileira de Pesquisas em Tuberculose, Rio de Janeiro, RJ, Brasil.; 3 Universidade Federal do Rio Grande, Rio Grande, RS, Brasil.; 4 Organização Pan-Americana de Saúde, Brasília, DF, Brasil.; 5 Universidad de la República, Facultad de Ciencias, Montevideo, Uruguay.; 6 Sociedad Latinoamericana de Tuberculosis y otras micobacteriosis. Uruguay.; 7 Instituto de Biología Molecular y Celular de Rosario, Santa Fe, Argentina.; 8 Universidade de São Paulo, Escola de Enfermagem de Ribeirão Preto, Centro Colaborador de la OPS/OMS para el Desarrollo de la Investigación en Enfermería, Ribeirão Preto, SP, Brasil.

En el último informe de la Organización Mundial de la Salud se subraya la alarmante situación de la tuberculosis (TB) en América Latina (AL). Entre 2020 y 2023 se observa en AL un aumento significativo de la mortalidad (14%) y un leve descenso en 2024 (13%)^(1)^. El presente artículo tiene como objetivo analizar esta problemática y destacar la importancia de conformar una Red de Investigación en TB en la región para alcanzar el objetivo de la OMS de poner fin a la TB en AL.

Los países de AL enfrentan múltiples desafíos que obstaculizan un progreso más acelerado hacia la eliminación de la TB. Estos incluyen factores socioeconómicos, culturales, educativos y de organización de los servicios de salud. Así, problemáticas como la coinfección TB/VIH, el aumento de la diabetes, el tabaquismo y otras enfermedades crónicas se suman a factores como la desnutrición, el consumo de sustancias psicoactivas y el acceso limitado a los servicios de salud. Factores que se ven acentuados por las desigualdades sociales y económicas de la región que hacen aún más difícil la eliminación de la TB en AL.

Otro aspecto preocupante es el incremento de casos de TB multirresistente (TB-MDR) y TB extremadamente resistente (TB-XDR), destacándose su distribución heterogénea, que varió en 2023 entre 0-11,9% del total de los nuevos casos de TB, según el país de AL^(1)^. Estos casos representan un desafío crítico para los sistemas de salud y para los pacientes debido al tratamiento prolongado, que puede superar los 20 meses, y al riesgo de transmisión de cepas resistentes. Aunque cabe señalar que en varios países de la Región ya se cuenta con los esquemas acortados orales BPaL/BPaLM de seis meses de duración para RR/MDR y preXDR.

Además, el impacto económico es considerable. Mientras que tratar un caso de TB sensible cuesta entre 100 y 500 dólares, el tratamiento de TB-MDR oscila entre 5.000 y 15.000 dólares, y para TB-XDR puede superar los 30.000 dólares por paciente^(2)^. Si bien la región puede contar con la ayuda del fondo estratégico de la Organización Panamericana de Salud para subsidiar los costos de estos tratamientos, aún queda mucho trabajo por hacer para que todos los pacientes puedan tener acceso. Este panorama refuerza la necesidad urgente de fortalecer las estrategias de prevención, diagnóstico precoz y manejo adecuado de la resistencia a medicamentos en la región.

El diagnóstico temprano de la TB en muchos países de AL enfrenta serias dificultades debido a la insuficiente disponibilidad de recursos para diagnósticos rápidos mediante técnicas de laboratorio o a través de imagenología y a la insuficiente capacitación de los profesionales de la salud. Sin embargo, es importante destacar que AL cuenta con los mejores niveles de confirmación bacteriológica a nivel global, alcanzando un promedio de 86% en 2023. Este indicador refleja la mejora de la capacidad de la región para realizar diagnósticos oportunos con los métodos actualmente disponibles, lo que representa un importante avance en la lucha contra la TB^(1)^. Aún así es necesario expandir la búsqueda activa enfocada en poblaciones en mayor situación de vulnerabilidad y zonas afectadas por la TB.

En 2023, durante la 78ª Asamblea General de las Naciones Unidas se aprobó la declaración política “Fin a la TB”, un compromiso global que destaca la necesidad de avanzar en ciencia, el financiamiento y la innovación, así como sus beneficios para combatir de manera urgente la epidemia mundial de TB. Esta declaración pone énfasis en garantizar el acceso equitativo a la prevención, pruebas diagnósticas, tratamiento y atención digna para todas las personas afectadas^(3)^. La Estrategia Fin a la TB, subraya el trabajo en Red como uno de los principales enfoques para alcanzar el objetivo de poner fin a la TB como epidemia para 2035 y eliminarla completamente para 2050^(4)^. El enfoque implica una colaboración integral e interdisciplinaria que reúne actores, como gobiernos, organizaciones no gubernamentales, la industria, profesionales de la salud, investigadores, gestores y la sociedad civil^(5)^.

El trabajo en red se estructura en torno a cuatro objetivos centrales^(5)^ que son: 1. Fortalecer la atención integral y centrada en las personas afectadas por TB; 2. Intensificar las políticas públicas y los sistemas de apoyo relacionados con la TB; 3. Fomentar la investigación e innovación para mejorar las herramientas diagnósticas, terapéuticas y preventivas; y 4. Asegurar la sostenibilidad financiera y el compromiso político a largo plazo para erradicar la enfermedad (Figura 1).

**Figura 1-:** Objetivos fundamentales de una Red Cooperativa de Tuberculosis

Fuente: https://new.aidsdatahub.org/sites/default/files/resource/toolkit-developing-national-tb-research-plan-2016.pdf

NTP = Programa Nacional de Tuberculosis; CSO = Organización de la sociedad civil; NGO = Organizaciones no gubernamentales; MoH = Ministerio de Salud

Se han descrito varias iniciativas a nivel internacional para cumplir con este propósito, entre estas se destacan:

**Iniciativa Europea de Investigación en Tuberculosis (ERI-TB):** Focalizada en garantizar la investigación y la innovación necesarias para la eliminación de la TB en Europa.

**Red de Investigación de TB BRICS:** Lanzada en 2017, esta red incluye a Brasil, Rusia, India, China y Sudáfrica, y más recientemente ha incorporado a Irán, Egipto y Etiopía.

**Tuberculosis Etíope y Comité Asesor de Investigación (TRAC):** Enfocados en fortalecer la capacidad de investigación y las estrategias para abordar la TB en contextos específicos.

**Centro Integrado de Investigación sobre Tuberculosis y Respirología de Vietnam (VICTORY):** Una iniciativa clave para la investigación y el desarrollo en Asia, orientada hacia la prevención y tratamiento de enfermedades respiratorias, incluida la TB.

Estas iniciativas reflejan el compromiso global en la lucha contra la TB, promoviendo la colaboración entre regiones y países para alcanzar soluciones innovadoras y efectivas^(5)^.

Si nos enfocamos en AL, existen antecedentes de cooperación regional. Por ejemplo, en Brasil destaca la iniciativa Red Brasileña de Investigación sobre Tuberculosis (REDE-TB), lanzada en 2001, como una iniciativa clave en la promoción de la investigación, colaboración científica y articulación con el programa nacional de control de la TB. Otras iniciativas similares que conectan la investigación y la clínica existen en Colombia, Chile, México y Perú, pero no hay una conexión entre ellas. Además, en 2006 fue consolidada la Sociedad Latinoamericana de Tuberculosis y otras micobacteriosis (SLAMTB), red de científicos de AL que ha contribuido a la promoción, información y colaboración científica entorno a la TB, sin mayor coordinación política con otros actores y estrategias para cumplir con los cuatro puntos centrales de una Red.

El establecimiento de una red de investigación amplia e integrada en AL es de esperar tenga un impacto significativo en el avance hacia la eliminación de la TB. Un ejemplo del valor de la colaboración científica es un estudio que demostró que los países con mayores índices de colaboración internacional son capaces de impulsar la cantidad y calidad de la producción científico-tecnológica^(6)^.

El intercambio de conocimiento no solo fomenta el trabajo conjunto entre investigadores, sino que también impulsa el desarrollo de métodos más eficaces para la prevención, diagnóstico y tratamiento de la TB. La falta de un enfoque integrado que combine actores estratégicos, como gobierno, academia y sociedad civil, dificulta el avance en la lucha contra la enfermedad y subraya la necesidad urgente de una red regional sólida y coordinada. Es imprescindible adoptar un enfoque integrado y coordinado que trascienda el sector de la salud, involucrando también a la educación, la participación comunitaria, las políticas sociales y la cooperación internacional.

Esta red colaborativa permitirá maximizar los recursos disponibles, mejorar el acceso equitativo a la atención, elevar la calidad de los servicios y promover la investigación y la innovación. De esta manera podremos crear un entorno propicio para la investigación e innovación en TB, incrementar el financiamiento para investigación e innovación en la región, promover y mejorar los enfoques para el intercambio de datos y garantizar el acceso equitativo a los beneficios de la investigación y la innovación. Bajo el respaldo de la Organización Panamericana de la Salud y con la participación de todos los países de la región, se fortalecerá la respuesta integral a la TB, respetando las particularidades y capacidades de cada nación. Este esfuerzo conjunto reafirma el compromiso de AL con un futuro donde la TB sea eliminada y la equidad en salud sea una realidad.
